# 31 days of COVID-19—cardiac events during restriction of public life—a comparative study

**DOI:** 10.1007/s00392-020-01681-2

**Published:** 2020-06-03

**Authors:** Manuel Rattka, Michael Baumhardt, Jens Dreyhaupt, Dietrich Rothenbacher, Kevin Thiessen, Sinisa Markovic, Wolfgang Rottbauer, Armin Imhof

**Affiliations:** 1 Clinic for Internal Medicine II, University Hospital Ulm-Medical Center, Albert Einstein Allee 23, 89081 Ulm, Germany; 2 Institute of Epidemiology and Medical Biometry, Ulm University, Ulm, Germany

**Keywords:** COVID-19, Cardiac events, Acute coronary syndrome, Epidemiology

## Abstract

**Aims:**

The coronavirus SARS-CoV-2 outbreak led to the most recent pandemic of the twenty-first century. To contain spread of the virus, many nations introduced a public lockdown. How the pandemic itself and measures of social restriction affect hospital admissions due to acute cardiac events has rarely been evaluated yet.

**Methods and Results:**

German public authorities announced measures of social restriction between March 21st and April 20th, 2020. During this period, all patients suffering from an acute cardiac event admitted to our hospital (*N* = 94) were assessed and incidence rate ratios (IRR) of admissions for acute cardiac events estimated, and compared with those during the same period in the previous three years (2017–2019, *N* = 361). Admissions due to cardiac events were reduced by 22% as compared to the previous years (*n* = 94 vs. an average of *n* = 120 per year for 2017–2019). Whereas IRR for STEMI 1.20 (95% CI 0.67–2.14) and out-of-hospital cardiac arrest IRR 0.82 (95% CI 0.33–2.02) remained similar, overall admissions with an IRR of 0.78 (95% CI 0.62–0.98) and IRR for NSTEMI with 0.46 (95% CI 0.27–0.78) were significantly lower. In STEMI patients, plasma concentrations of high-sensitivity troponin T at admission were significantly higher (644 ng/l, IQR 372–2388) compared to 2017–2019 (195 ng/l, IQR 84–1134; *p* = 0.02).

**Conclusion:**

The SARS-CoV-2 pandemic and concomitant social restrictions are associated with reduced cardiac events admissions to our tertiary care center. From a public health perspective, strategies have to be developed to assure patients are seeking and getting medical care and treatment in time during SARS-CoV-2 pandemic.

**Electronic supplementary material:**

The online version of this article (10.1007/s00392-020-01681-2) contains supplementary material, which is available to authorized users.

## Introduction

Sudden, unforeseen, or stressful events, like natural disasters or sports finals that affect a large number of people in defined areas, are known to potentially increase the risk of cardiac events including cardiac arrhythmia, acute coronary syndrome, and even out-of-hospital cardiac arrest [1–3].

Spread of the novel coronavirus (SARS-CoV-2) resulted in a rapidly expanding global pandemic with substantial regional differences in overall infection numbers, the number of infected getting COVID-19 disease and requiring medical treatment, and the percentage of those in severe condition requiring treatment in an intensive care unit. Recently, it has been reported that in Austria, hospital admissions due to acute coronary syndrome declined significantly during March 2020, suggesting a potential undersupply of patients with cardiac disease during the COVID-19 outbreak [4]. As shown in a very recent report from Northern Italy, a region hit very hard by the pandemic, hospital admissions due to acute coronary syndrome (ACS) were significantly reduced as compared to the months before the pandemic as well as the same period in the previous year [5].

In Germany, the first known SARS-CoV-2 infections were confirmed in late January 2020. Extensive public health measures were initiated on March 21st, 2020 to minimize people’s physical contact to avoid further spread of SARS-CoV-2 and to control the COVID-19 outbreak and initially scheduled to last until the April 20^th^, 2020. However, social isolation can also have detrimental effects on the people’s health, but the cardiovascular consequences of these measures have rarely been studied yet [6].

This study sought to investigate the hospital admissions due to acute cardiac events including acute coronary syndrome, cardiac arrhythmia, and out-of-hospital cardiac arrest in a tertiary care center in the South of Germany during the national lockdown including social restrictions in comparison to the previous years when no pandemic crisis was announced. Moreover, we compared markers of myocardial damage in patients presenting with ST-segment elevation (STEMI) to assess potential delay of admission.

## Methods

### Study design and participants

This retrospective comparative study included all consecutive patients who were admitted to either the emergency department (ED) or the intensive care unit (ICU) at Ulm University Hospital with one of the final clinical discharge diagnoses: (1) acute coronary syndrome (ACS) comprising myocardial infarction with ST-segment elevation (STEMI), myocardial infarction without ST-segment elevation (NSTEMI), and unstable angina (UA), (2) cardiac arrhythmia, and (3) out-of-hospital cardiac arrest. All collected data were part of routine assessment and routine procedures conducted during the hospital stay, no study specific procedures were involved. All ACS patients underwent a percutaneous coronary intervention (PCI) because of severe coronary artery stenosis or total occlusion, as indicated by the current guidelines [7, 8]. The study period comprises 31 days between March 21st, 2020, when rigorous public health measures came into effect, and April 20th, 2020, when public opening was partly initiated. A number of patients admitted to our hospital with acute cardiac events were assessed daily in a retrospective manner and compared with the same periods in the previous three years (2017–2019). Only patients admitted from the region usually covered by our hospital by emergency medical services were included. Patients transferred from other hospitals were excluded. The study complies with the Declaration of Helsinki and was approved by the local ethics committee (number of application and positive vote 130/20).

### Data collection

Demographic, clinical, laboratory, and outcome data were extracted from electronic medical records by two physicians separately (MR und MB) and adjudicated by a third one (AI) in case of any kind of difference.

### Definitions

**Fig. 1:**
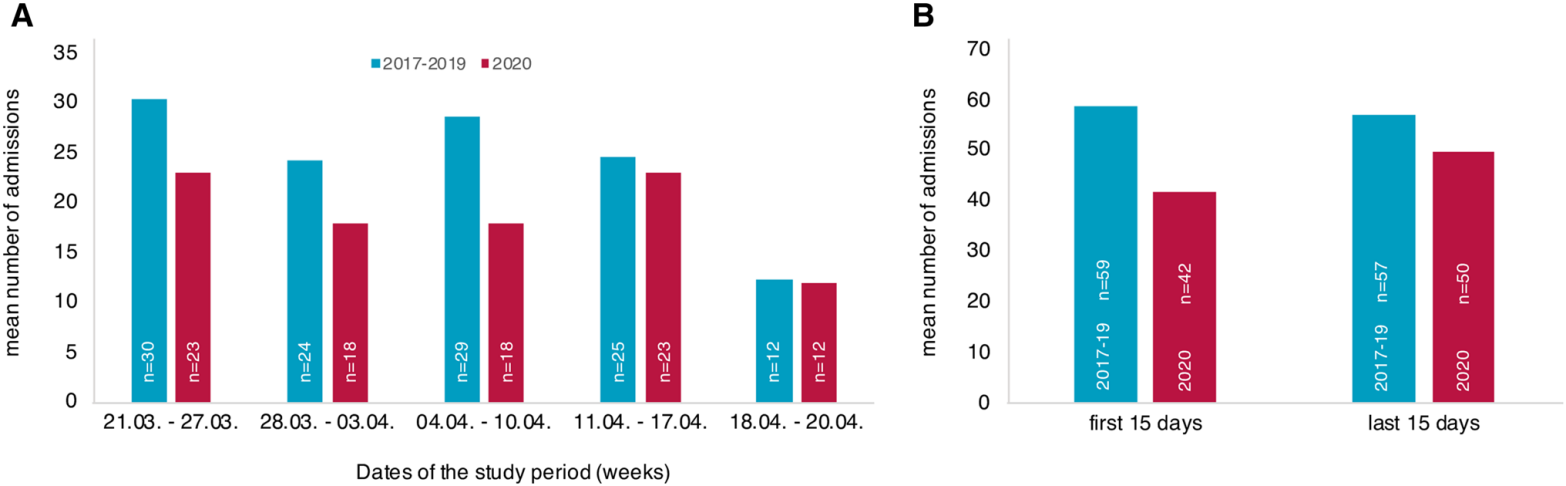
Mean number of admitted patients. Mean number of patients per week admitted with cardiovascular events in 2020 (red) and 2017–2019 (blue) (**a**), and in the first and the last 15 days of the study (blue) and control period (red) (**b**)

Classification in STEMI, NSTEMI, and UA was done by clinical presentation, reviewing the electrocardiogram report, high-sensitivity troponin T (hsTnT) plasma concentrations at admission, and in compliance with the current guidelines [7, 8]. To avoid inclusion bias, patients classified to suffer from unstable angina were only included in the analysis when they finally underwent cardiac catheterization with percutaneous coronary intervention [7]. Arrhythmic events had to be documented at the time of admission by the hospital staff, by the admitting emergency medical service, or by an implanted cardiac device. Absolute numbers of admission in Fig. 1 were reported on a weekly basis from Saturday (March 20th) to Friday and the remaining three days till the ending of complete restrictions (1a) and according to the first and the last 15 days of the study period (1b).

### Laboratory procedures

Blood samples were drawn at the time of hospital admission for measurements of hsTnT (ElectroChemiLumineszenz ImmunoAssay “ECLIA” Roche, Cobas 8000), creatine kinase (CK), and lactate dehydrogenase (LDH) as part of the clinical routine. Also, every patient was tested for SARS-CoV-2 by throat swab test (Sigma-Virocult^®^ with 2 ml Virocult^®^ medium, Check Diagnostics GmbH, Germany) and analyzed by PCR at the local Institute for Virology.

### Statistical analysis

Continuous variables were presented as median and interquartile range (IQR) or mean and standard deviation as appropriate, categorical variables as absolute and relative frequencies, respectively. Mann–Whitney *U* test, Chi^2^ test, or Fisher’s exact test were used to compare variables between study period and control period where appropriate. Crude incidence rates per day and incidence rate ratios including 95% confidence intervals (CI) comparing the study period with the control periods were calculated by the use of Poisson regression to model the number of hospitalizations per day. Statistical analyses were performed with SAS version 9.4 under Windows. A two-sided *p* value of 0.05 was considered to be statistically significant. Owing to the explorative nature of this study, all the results from statistical tests have to be interpreted as hypothesis generating. An adjustment for multiple testing was not done.

## Results

During the study period and the comparative periods, a total of 455 patients with acute cardiac events were admitted to Ulm University Hospital and included into the study (Table 1). When comparing the study period of 2020 to the control periods 2017–19, 33% and 37% were females (*p* = 0.46) and patients were slightly younger with 67 ± 13 and 70 ± 12 years, respectively (*p* = 0.07). At least one cardiovascular risk factor was present in 91% of patients (*n* = 416) of which arterial hypertension (79%) and dyslipidemia were the most frequent (62%). 45% of all patients had known coronary artery disease and 9% a history of TIA or stroke. Mean heart rate at admission was 92 ± 30/min and systolic blood pressure 138 ± 26 mmHg without significant differences between the groups. Most of the baseline characteristics of patients in 2020 were similar to those in 2017–2019 except for history of TIA or stroke (15% vs. 7%, *p* = 0.02), dyslipidemia (52% vs. 64%, *p* = 0.03), and diastolic blood pressure (85 ± 14 mmHg vs. 77 ± 16 mmHg, *p* = < 0.01), and are summarized in Table 1. None of the 2020 cohort was tested positive for SARS-CoV-2 infection by PCR.

**Table 1:** Demographics and clinical characteristics

	Total (*n* = 455)	Study period (*n* = 94)	Control period (*n* = 361)	*p* value
Female sex	165 (36%)	31 (33%)	134 (37%)	0.46
Age (years)	69 ± 12	67 ± 13	70 ± 12	0.07
Heart rate at admission (bpm)	92 ± 30	94 ± 27	92 ± 30	0.20
Blood pressure systolic (mmHg)	138 ± 26	135 ± 18	138 ± 28	0.53
Blood pressure diastolic (mmHg)	79 ± 15	85 ± 14	77 ± 16	< 0.01
Known cardiac arrhythmia	174 (38%)	30 (32%)	144 (40%)	0.15
Known CAD	203 (45%)	40 (43%)	163 (45%)	0.64
Arterial hypertension	360 (79%)	77 (82%)	283 (79%)	0.48
Dyslipidemia	281 (62%)	49 (52%)	232 (64%)	0.03
Diabetes mellitus	109 (24%)	21 (22%)	88 (24%)	0.67
Family history	98 (22%)	20 (21%)	78 (22%)	0.93
Obesity	60 (13%)	13 (14%)	47 (13%)	0.85
Smoker	153 (34%)	33 (35%)	120 (33%)	0.75
OSAS	19 (4%)	5 (5%)	14 (4%)	0.54
COPD	25 (6%)	7 (7%)	18 (5%)	0.35
History of TIA/stroke	40 (9%)	14 (15%)	26 (7%)	0.02

The data are mean ± standard deviation or absolute frequencies (percent)

bpm, beats per minute; CAD, coronary artery disease; OSAS, obstructive sleep apnea syndrome; COPD, chronic pulmonary obstructive disease

Figure 1a shows the mean numbers of acute cardiac events per week and Fig. 1b the number of admissions in the first 15 days and the last 15 days during the periods in 2017–19 and in 2020. In 2020, overall fewer patients (− 22%) were admitted to our hospital as compared to the previous years. Moreover, admissions dropped during the first 15 days as compared to the last 15 days of the study period (42 vs. 50 patients) indicating an acute effect when public restrictions came to effect. This differed from the constant rates in the previous years (59 vs. 57 patients).

A number of hospital admissions due to acute cardiac events and laboratory findings in patients presenting with STEMI are summarized in Table 2 (and in detail in the Online Resource 1). ACS represents the major diagnosis in both periods (55% in 2020 and 57% in 2017–19). Distribution of diagnoses did not differ between the study and the control period. In order to assess whether the COVID-19 outbreak and the public restrictions potentially delayed admission of patients even with STEMI, we compared measured hsTnT, CK, and LDH plasma concentrations at admission. We observed that initial hsTnT concentrations were significantly higher in patients during the SARS-CoV-2 pandemic in 2020 (644 ng/l, IQR 372–2388) as compared to those admitted in 2017–2019 (195 ng/l, IQR 84–1134; *p* = 0.02) indicating a prolonged time between the onset of symptoms and first medical contact.

**Table 2:** Hospital admissions due to acute cardiac events

	Total	Study period	Control period	*p* value
Overall admissions	455	94	361	0.15
Acute coronary syndromes	259 (57)	52 (55)	207 (57)	
STEMI	56 (12)	16 (17)	40 (11)	
NSTEMI	120 (26)	16 (17)	104 (29)	
Unstable angina	83 (18)	20 (21)	63 (17)	
Out-of-hospital cardiac arrest	28 (6)	6 (7)	22 (6)	
Cardiac arrhythmia	168 (37)	36 (38)	132 (37)	
*Plasma concentration of biomarkers of myocardial damage at hospital admission in STEMI*				
Troponin T (ng/l)	375 (129–1616)	644 (372–2388)	195 (84–1134)	0.02
Creatine kinase (U/l)	271 (151–1039)	418 (184–1357)	243 (115–1039)	0.25
Lactate dehydrogenase (U/l)	236 (190–295)	208 (192–273)	251 (190–295)	0.56

The data are absolute frequency (percent) or median with inter-quartile range (IQR)

STEMI, ST-segment elevation myocardial infarction; NSTEMI, non-ST-segment elevation myocardial infarction

Table 3 shows the crude incidence rate (per day) and incidence rate ratios for acute cardiac events. Overall admissions due to cardiac events during the COVID-19 outbreak were significantly lower as compared to 2017–19 (IRR 0.78; 95% CI 0.62–0.98). Admissions due to all ACS declined as well, but the 95% CI included the null-effect value (IRR 0.75; 95% CI 0.56–1.02). Of note, significantly fewer patients with NSTEMI were admitted (IRR 0.46; 95% CI 0.27–0.78). In contrast, we did not observe a significant decrease in admissions due to unstable angina, STEMI, cardiac arrhythmia, and out-of-hospital cardiac arrest.

**Table 3:** Incidence rate ratios of hospital admissions

Group	Study period (*n* = 94)	Control period (*n* = 361)
Overall		
No. daily admissions	3.032	3.882
IRR (95% CI)		0.78 (0.62–0.98)
*p* value		0.03
Acute coronary syndrome		
No. daily admissions	1.677	2.226
IRR (95% CI)		0.75 (0.56–1.02)
*p* value		0.07
STEMI		
No. daily admissions	0.516	0.430
IRR (95% CI)		1.20 (0.67–1.18)
*p* value		0.54
NSTEMI		
No. daily admissions	0.516	1.118
IRR (95% CI)		0.46 (0.27–0.78)
*p* value		< 0.01
Unstable angina		
No. daily admissions	0.645	0.677
IRR (95% CI)		0.95 (0.58–1.57)
*p* value		0.85
Out-of-hospital cardiac arrest		
No. daily admissions	0.194	0.237
IRR (95% CI)		0.82 (0.33–2.02)
*p* value		0.66
Cardiac arrhythmia		
No. daily admissions	1.161	1.419
IRR (95% CI)		0.82 (0.57–1.18)
*p* value		0.29

IRR, incidence rate ratio

## Discussion

In this comparative study, we deliver evidence for a significant decline in admission rates due to acute cardiac events in a regional, but representative sample of the German population as compared to the previous years within the time period of the national lockdown during the SARS-CoV-2 pandemic in 2020. Right from the onset of social restrictions, admission numbers strongly decreased. While admissions of patients with STEMI and out-of-hospital cardiac arrest remain similar, significantly fewer patients with NSTEMI have been admitted. Moreover, in patients suffering from STEMI, we observed significantly higher hsTnT concentrations at admission during the study period compared to the previous years, suggesting that affected patients suffer a delay, or avoid hospital admission, and subsequently may increasing their risk of adverse outcomes. Iatrophobia, the fear of getting infected while in the hospital and framing issues due to public calls to stay at home might be among major reasons for this observation.

After severe acute respiratory syndrome coronavirus (SARS-CoV) in 2003 and the Middle East respiratory syndrome coronavirus (MERS-CoV) in 2012, the severe acute respiratory syndrome coronavirus in 2019 (SARS-CoV-2) is the third coronavirus pandemic of the twenty-first century [9]. However, in contrast to the other two diseases, SARS-CoV-2 rapidly spread across the globe, infecting millions of people, and threatening their health and lives, and puts us globally in an unparalleled crisis situation. As a consequence, many countries initiated a global lockdown, including rigorous restrictions of public life to contain further spread of the virus, accompanied by daily crisis news in the media.

In Germany, many government decrees were implemented from March 21st, 2020 in order to minimize physical contact between people. Several public facilities, such as schools, universities, sports grounds, stadiums, amusement parks, cultural institutions, and shopping centers were closed. In addition, people were encouraged to work from home. The contact of more than two persons outside families was prohibited and a distance of 1.5 m between two persons had to be ensured. The pandemic and concomitant restrictions led to reduced working hours, rising unemployment rates, a stock market decline, and the fear of a global economic depression. Whether these measures also affect the incidence of acute cardiac events during a pandemic has rarely been evaluated yet and warrants further investigation.

Very recently, it has been shown for Northern Italy that the rate of admissions for acute coronary syndromes was significantly reduced during the COVID-19 outbreak and public shutdown to the same extent in patients with STEMI and NSTEMI [5]. Similar observations have also been reported from the United States of America, Spain, and Austria [4, 10, 11]. These findings are in line with our study on a subset of the German population for overall admission and NSTEMI, respectively, even in situations where intensive care capacities for COVID-19 patients were sufficient.

The decline in admission of patients with acute cardiac events might be attributable to several factors. The first possible factor is the numerous calls by the government and throughout various media (i.e., press news, television, and social media) for people to seek medical contact only in case of an emergency to avoid exceedance of the country’s health system performance capability. However, neither government officials nor the media clearly defined what would be considered an emergency. As a consequence, patients may have delayed first medical contact and misgauged the severity of their symptoms.

The second possible factor contributing to our findings is framing issues. As a result, chest pain and dyspnea might be primarily attributed to a respiratory infection, rather than ACS, by the patient or even by the attending medical emergency service [12].

Next, it is well known that physical and emotional stress are associated with the incidence of acute cardiac events [1–3, 13]. Both of which alone or in combination potentially affect every single patient in this specific situation. On one side measures of social distancing resulting in work at home and thus possibly a decrease in work stress, reduced participation in sports events, and a decrease in adverse work-related physical exercise, and might reduce the stress burden of daily life, and consequently cardiac events in parts of the general population [2, 14, 15]. On the other side, however, the imminent threat of unemployment and the fear of getting infected with the coronavirus might also increase the stress levels leading to a higher rate of cardiac events among other parts of the population [13]. We observed a significant decrease in patients admitted with NSTEMI, but similar or even slightly higher numbers of STEMI, suggesting that reduced stress burden did not contribute to the overall decline in acute cardiac events.

Finally, we believe iatrophobia might represent a major reason for the decrease in admissions due to acute cardiac events. Iatrophobia, which is defined as the fear of physicians, medical care, or the medical care system, is an important factor in delaying seeking care for medical conditions and their treatment [16]. Among others, iatrophobia comprises the patient’s fear of illness and medical examination, fear of physician reactions, and fear related to barriers of care [16]. Currently, the fear of getting in contact with SARS-CoV-2 infected patients and the risk of getting infected presumably keeps symptomatic patients from seeking acute medical care. Based on the personal experience, far more patients than before seek first medical contact by telephoning the physician on duty at our emergency department to report their symptoms and ask for advice on how to proceed. Moreover, some of them spontaneously admit a fear of getting infected while in the hospital. This hypothesis is supported by our observation that cardiac events usually accompanied by bearable symptoms and stable hemodynamic situations, such as NSTEMI declined while admissions due to STEMI, presumably felt more threatening by the patients, did not change. We assume that, due to iatrophobia, patients endure symptoms longer before seeking emergency medical advice. This puts them at risk for an adverse outcome, especially if those with ACS stay untreated [17]. A very recent study from Italy reported on a substantially increased STEMI case fatality rate, which might be attributable to a delay in presentation for urgent medical attention because of reasons aforementioned [18]. Additionally, in the Lombardy, a region in Italy struck hard by the SARS-CoV-2 pandemic, an increase in out-of-hospital cardiac arrest of 58% has been observed, while in our study, the IRR for out-of-hospital cardiac arrest remained the same [19]. Regional differences in COVID-19 burden including direct effects of the pandemic might partly explain this difference.

Compared with the previous years, in our study, we presumed delayed admissions even of STEMI patients with a substantially longer time to first medical contact (as well as prolonged pain to balloon time), potentially resulting in more complications afterwards [20]. This hypothesis is supported by our findings of significantly higher concentrations of hsTnT at admission in patients with STEMI compared with the previous years.

Our study has some limitations. This single center retrospective cohort study carries all the inherent limitations of retrospective research. However, as SARS-CoV-2 rapidly spread worldwide, the possibility to conduct a prospective study on the incidence of acute cardiovascular events has been, and is still very limited. Despite the retrospective character of our study, we closely assessed the patients admitted to our hospital with cardiovascular and other cardiac events daily from March 21^st^, 2020 until April 20th, 2020, and similar routine procedures as in the control period were applied. Thus, we believe that not a single event in the lockdown period has been missed. As a consequence, however, the decrease in admissions in 2020 as compared to 2017–2019 is probably underestimated. Furthermore, we can only hypothesize about the cause of the declined incidence, using clinical biomarkers, final diagnosis, and place of admission as surrogates. Questionnaires evaluating patients’ behaviors and attitudes were not available in time and should be part of future research. Moreover, to further substantiate our hypothesis of delayed admission of ACS patients, additional factors, including long-term mortality should be taken into consideration [21]. However, our study compared the current data with the previous years’ using similar diagnostic methods and thus allows us to profoundly quantify the effects in this population during similar and good comparable time periods.

## Conclusions

In conclusion, the SARS-CoV-2 pandemic is associated with a substantial reduction of admissions to our tertiary care hospital in the South of Germany due to acute cardiac events. Iatrophobia and framing issues, such as calls to strictly follow regulations for social distancing as to not overwhelm the healthcare system and stay at home messages may be among the major reasons for this observation. Further studies should investigate the possible effects on medical care, adverse outcomes, and potential excess morbidity and mortality in patients with cardiac disease. From a public health perspective, strategies have to be developed to maintain public awareness for life-threatening diseases, such as acute coronary syndromes to ensure adequate and timely medical care for all patients in order to prevent the potential additional excess morbidity and mortality not caused by COVID-19 itself, but other factors, including fear of getting infected and shutdown measures during future pandemics and at the present crisis.

## Electronic supplementary material

Below is the link to the electronic supplementary material.

Supplementary file1 (DOCX 12 kb)
