# Supplementary material for: 31 days of COVID-19—cardiac events during restriction of public life—a comparative study
Source: Clin Res Cardiol. 2020 Jun 3;109(12):1476–82. doi: 10.1007/s00392-020-01681-2 (PMC7268583; doi:10.1007/s00392-020-01681-2)
Supplement: Supplementary file 1 — Supplementary file1 (DOCX 12 kb) [file 392_2020_1681_MOESM1_ESM.docx]

| Online Resource 1: Hospital admissions due to acute cardiac events per years of the study period | | | | |
| --- | --- | --- | --- | --- |
|  | 2017 | 2018 | 2019 | 2020 |
| Overall admissions | 111 | 135 | 115 | 94 |
| Acute coronary syndromes | 69 (62) | 73 (54) | 65 (57) | 52 (55) |
| STEMI | 7 (6) | 16 (12) | 17 (15) | 16 (17) |
| NSTEMI | 35 (32) | 37 (27) | 32 (28) | 16 (17) |
| Unstable angina | 27 (24) | 20 (15) | 16 (14) | 20 (21) |
| Out-of-hospital cardiac arrest | 7 (6) | 8 (6) | 7 (6) | 6 (6) |
| Cardiac arrhythmia | 35 (32) | 54 (40) | 43 (37) | 36 (38) |
| Data are absolute frequency (%) | | | | |
